# Supplementary material for: Splice-Junction-Based Mapping of Alternative Isoforms in the Human Proteome
Source: Cell Rep. Author manuscript; Available in PMC 2020 Jan 15. (PMC6961840; doi:10.1016/j.celrep.2019.11.026)
Supplement: 2 [file NIHMS1546469-supplement-2.zip › DF1/PXD010154/Testis/Testis_43_EGFL7_GDTCQSGVDSAMKEEVQR.pdf]

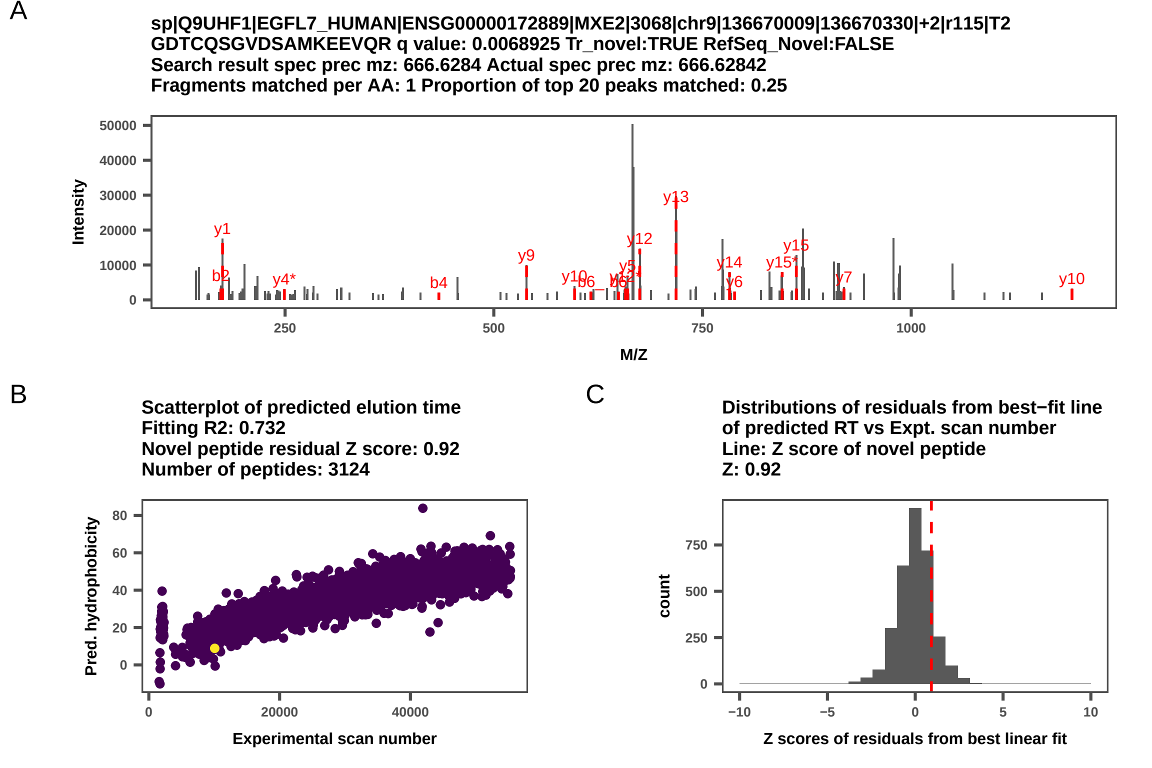
A
sp|Q9UHF1|EGFL7_HUMAN|ENSG00000172889|MXE2|3068|chr9|136670009|136670330|+2|r115|T2
GDTCQSGVDSAMKEEVQR q value: 0.0068925 Tr_novel:TRUE RefSeq_Novel:FALSE
Search result spec prec mz: 666.6284 Actual spec prec mz: 666.62842
Fragments matched per AA: 1 Proportion of top 20 peaks matched: 0.25
Intensity
50000
40000
30000
20000
10000
0
y1
b2
y4*
b4
y9
y12
y13
y14
y15
y15*
y6
y7
y10
M/Z
250
500
750
1000
B
Scatterplot of predicted elution time
Fitting R2: 0.732
Novel peptide residual Z score: 0.92
Number of peptides: 3124
Pred. hydrophobicity
80
60
40
20
0
Experimental scan number
0
20000
40000
C
Distributions of residuals from best−fit line
of predicted RT vs Expt. scan number
Line: Z score of novel peptide
Z: 0.92
count
750
500
250
0
Z scores of residuals from best linear fit
−10
−5
0
5
10
